# Randomly polarised beam produced by magnetooptically Q-switched laser

**DOI:** 10.1038/s41598-017-15826-3

**Published:** 2017-11-13

**Authors:** Ryohei Morimoto, Taichi Goto, Takunori Taira, John Pritchard, Mani Mina, Hiroyuki Takagi, Yuichi Nakamura, Pang Boey Lim, Hironaga Uchida, Mitsuteru Inoue

**Affiliations:** 1 Department of Electrical and Electronic Information Engineering, Toyohashi University of Technology, 1-1 Hibari-Ga-Oka, Tempaku, Toyohashi, Aichi 441-8580 Japan; 2 JST, PRESTO, 4-1-8 Honcho, Kawaguchi, Saitama, 332-0012 Japan; 3 Institute for Molecular Science, Laser Research Centre, 38 Nishigonaka, Myodaiji, Okazaki, Aichi 444-8585 Japan; 4 Electrical and Computer Engineering Department, Iowa State University, Ames, Iowa 50011 USA

## Abstract

Diode-pumped solid-state micro lasers are compact (centimetre-scale), highly stable, and efficient. Previously, we reported Q-switched lasers incorporating rare-earth substituted iron garnet (RIG) film. Here, the first demonstration of the magnetooptical (MO) Q-switch in an Nd:YAG laser cavity is performed. We fabricate a quasi-continuous-wave (QCW) diode-pumped Nd:YAG laser cavity, which is shortened to 10 mm in length and which contains an RIG film and a pair of small coils. This cavity yields a 1,064.58-nm-wavelength pulse with 25-ns duration and 1.1-kW peak power at a 1-kHz repetition ratio. Further, the polarisation state is random, due to the isotropic crystal structure of Nd:YAG and the fact that the MO Q-switch incorporating the RIG film does not require the presence of polarisers in the cavity. This is also the first report of an MO Q-switch producing random polarisation.

## Introduction

Since their initial development, lasers have been implemented as irreplaceable components of various applications, e.g., mass spectrometers^1^, laser machining devices^2–5^, car ignition plugs^6,7^, satellite propulsion devices^8–10^, and medical equipment^11,12^. In particular, the Q-switching technique allows solid-state lasers to generate a short, high-powered pulse output^13–15^, which is crucial for the above applications.

Previously, we proposed a magnetooptical (MO) Q-switch composed of a ferrimagnetic rare-earth substituted iron garnet (RIG) film and small coils^16,17^. Magnetic garnets are well known for their large MO effects and high transmittance in the near-infrared region^18,19^. However, although RIGs have potential use in optical applications such as two-dimensional integrated arrays^20^ and storage media^21^, there are few reports of Q-switches employing these materials. In one of our previous studies, we demonstrated the MO Q-switch in a diode-pumped Nd:GdVO_4_ laser system and showed the potential of this Q-switch, for which a notably compact system size was obtained (cavity length *L*: 10 mm). Note that such a small *L* is impossible using other active Q-switches, for example, electro-optic (EO)^22^ and acousto-optic (AO)^23^ Q-switches. EO Q-switches require a cubic polariser in the cavity and a high-voltage power supply for operation, whereas the AO module in an AO Q-switch cannot be made appropriately small to yield sufficient interaction and radio-frequency (RF) power supply for operation. Therefore, it is difficult to miniaturise *L* or the entire laser system for these Q-switches. As higher output photon densities can be achieved for Q-switched lasers with shorter length *L* ^14,24^, diode-pumped solid-state micro lasers having *L* values of millimetre order are attracting interest.

Although the compactness of the MO Q-switched laser incorporating RIG film was demonstrated previously, the output peak power remained small. Therefore, in this paper, a quasi-continuous-wave (QCW)^25,26^ pumping technique using pulsed pumping light is employed to provide higher pump energy to the lasing material. In addition and for the first time, Nd:YAG emitting randomly polarised light is used as a laser material to demonstrate the MO Q-switch. The combination of Nd:YAG and an MO Q-switch has special meaning unlike other Q-switches because a common misunderstanding is that the Q-switching using MO materials is only based on the Faraday rotation, meaning that the input light must always be in a linearly polarised state while using the MO Q-switch. Regarding the achievement of integrated actively Q-switched micro lasers, Nd:YAG is a promising lasing material. The crystal structure of Nd:YAG is similar to RIG, and these materials have similar thermal expansion coefficients^27–30^; thus, RIG film can grow on the Nd:YAG via epitaxial growth techniques^31^ or bond to the Nd:YAG surface as Cr^4+^:YAG^32,33^. The latter material also possesses a garnet structure and a similar thermal expansion coefficient, and has been reported as an appropriate material for a passive Q-switch with high miniaturisation^34,35^. Therefore, the performance of MO Q-switching using RIG in an Nd:YAG laser is important for the implementation of actively Q-switched micro lasers. Finally, the use of a different lasing material also facilitates discussion of the polarisation state of the MO Q-switched laser. The RIG-based Q-switch does not require the presence of a polariser in the cavity; therefore, the isotropic structure of the Nd:YAG must affect the output polarisation state.

## Experimental setup

**Figure 1:**
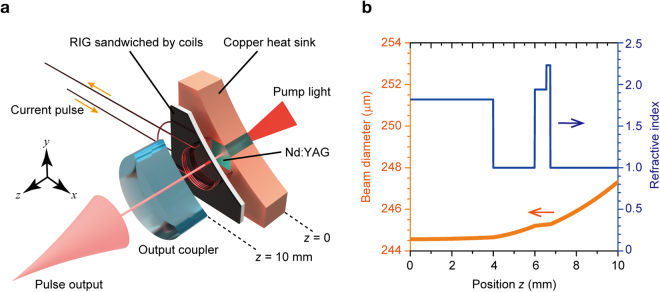
Diode-pumped magnetooptical (MO) Q-switched laser. (**a**) Schematic of laser cavity setup. The rare-earth substituted iron garnet (RIG) film was sandwiched by a pair of coils and its magnetisation was modulated by a pulsed field. The minimum cavity length *L* was 10 mm. (**b**) Beam diameter (thick orange line) estimated using ABCD matrix method and refractive indexes (thin blue line) in cavity.

A schematic of the prepared Q-switched laser cavity is shown in Fig. 1a, with parts of the cavity components being cut away for improved visibility. A diode at 808-nm wavelength end-pumped the 0.5 at.% Nd:YAG crystal, which was 3 mm × 3 mm × 4 mm in size. The Nd:YAG crystal was wrapped in foil and fixed in the water-cooled Cu heat sink, and its temperature was stabilised at 20 °C by a proportional-integral-derivative-controlled Peltier cooler. Dielectric multilayer coating was present on the input and output surfaces of the Nd:YAG, having high reflectance (HR) of 99.8% at the 1,064-nm wavelength and high transmittance (HT) of 98% at 808-nm wavelength on the input side, and 98% HT and 99.8% HR at 1,064- and 808-nm wavelengths, respectively.

A concave mirror with 300-mm curvature radius and 90% reflectance at the 1,064-nm wavelength was placed 10-mm from the Nd:YAG input surface as an output coupler. The MO Q-switch was inserted in the cavity. The RIG used in this study was the same 190-μm-thick film employed in our previous studies^16,17^, which was formed via a liquid-phase epitaxy method on a 560-μm-thick single-crystalline Gd_3_Ga_5_O_12_ substrate. The composition of this film measured by energy-dispersive x-ray spectroscopy (JEOL, JED-2201F) was Tb_2.0_Bi_1.0_Fe_4.8_Ga_0.2_O_12–ξ_, where ξ indicates the number of oxygen vacancies. The optical loss characterized by a spectrophotometer (UV-3150, Shimadzu) was 108 dB/cm at the wavelength of 1,064 nm. The Faraday rotation angle at 1,064-nm wavelength measured via the rotating analyser method was 2.4 × 10^3^ °/cm. Further, the figure of merit (FOM) defined by the Faraday rotation angle divided by the absorption was 222 °/dB. Maze-shape magnetic domains appeared in the RIG with an average width of ~50 μm. A pair of small coils with 5.3-mm diameter sandwiched the RIG and were connected to a pulse current generator, which applied a peak current of 56 A with 3-μs duration for the applied pulsed field. The generated field applied to the coil was estimated to be more than 200 Oe, which is the saturation field of the RIG film.

Figure 1b shows the beam diameter in the cavity estimated using the ABCD matrix method^24^, along with the refractive indexes of the cavity components. The beam diameter in the RIG was approximately 245 μm, which is five times larger than the average widths of the magnetic domains in the RIG film. The laser output was simultaneously monitored by an energy meter (Ophir, VEGA) and an InGaAs-based fast-response optical detector (Thorlabs, DET10C/M).

## Results

### Peak power and beam quality

The pulse width *τ*_p_ and peak power of a Q-switch laser are proportional and inversely proportional to *L*, respectively^36^. The relation between *τ*_p_ and *L* is expressed as^14^

1 $${\tau }_{p}\approx \frac{r\eta (r)}{r-1-\,\mathrm{ln}\,r}{\tau }_{c}=\frac{r\eta (r)}{r-1-\,\mathrm{ln}\,r}(\frac{2L}{c\delta }),$$

where *r* is the inversion ratio, *η* is the energy extraction efficiency, *τ*_c_ is the cavity delay time, *c* is the velocity of light, and *δ* is the cavity loss before Q-switching. The values of *c*, *r*, *η*, and *δ* were 3.0 × 10^8^ m/s, 1.063, 0.908, and 1.127, respectively. These were derived by the relationships *r* = *N*_i_/*N*_th_, *η* = (*N*_i_ − *N*_f_)/*N*_i_, *δ* = −ln(*R*) − 2ln(*T*_on_), where *N*_i_ is the initial population inversion density, *N*_th_ is the threshold population inversion density, *N*_f_ is the final population inversion density, *R* is the reflectance of the output coupler (=0.9), and *T*_on_ is the transmittance of the RIG film when the field was applied (=0.582). Moreover, the values of *N*_i_, *N*_th_, and *N*_f_ are determined by the equations *N*_i_ = (*δ* + *δ*_Q_)/(2*σL*), *N*_th_ = *δ*/(2*σL*), and *N*_f_ = *N*_i_ − *N*_th_·ln(*N*_i_/*N*_f_), where *σ* is the stimulated emission cross-section of Nd:YAG ( = 2.8 × 10^−23^ m^2^)^14^ and *δ*_Q_ is the additional loss caused by the Q-switch. The peak power is equivalent to *E*_o_/*τ*_p_, where *E*_o_ is the output energy.

**Figure 2:**
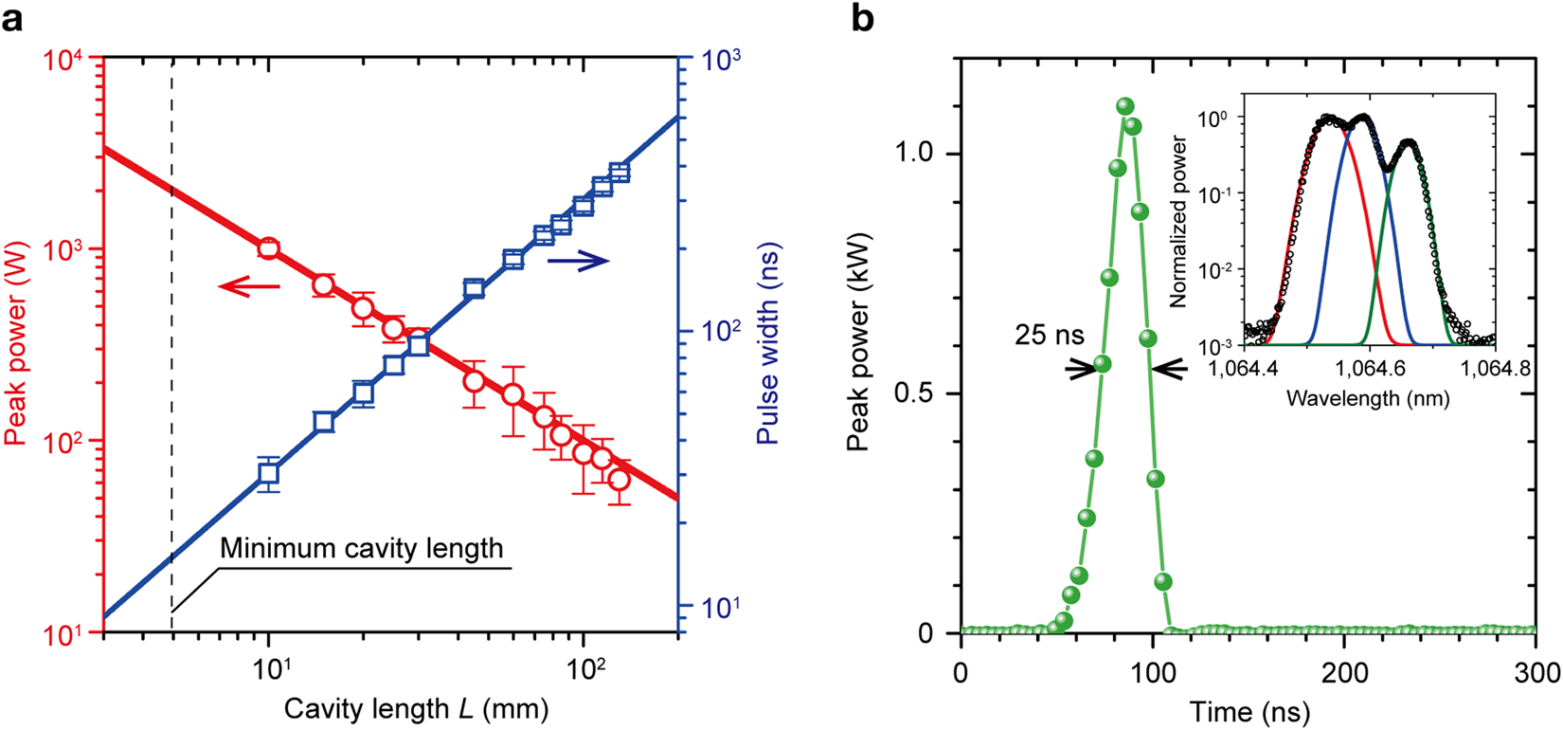
Output pulse characteristics. (**a**) Peak power and pulse width for varying *L*. Pumping energy: 6.4 mJ/pulse, corresponding pumping duration: 200 μs. The solid lines indicate the calculated values, which agree well with the measured values. The thin dashed line indicates the minimum *L* achievable using the Nd:YAG and RIG film. (**b**) Output pulse shape with *L* = 10 mm. The peak power and pulse duration were 1.1 kW and 25 ns, respectively. Inset: output wavelength spectrum. Black circles show the measured data. Three peaks were observed at the wavelength of 1,064.54 (red line), 1,064.59 (blue line), and 1,064.66 nm (green line). The spectrum widths at each wavelength were 47, 39, and 35 pm, respectively.

To examine the influence of *L* on the peak power and beam quality, the output coupler was gradually moved along the beam propagation axis to vary *L* from 10 to 130 mm. The pumping repetition ratio and pump duration were fixed to 1 kHz and 200 μs, respectively, corresponding to a pumping energy of 6.4 mJ/pulse, because the obtained peak power was maximum in our setup. Figure 2a shows the peak power and *τ*_p_ results for varying *L*. The obtained values (circles and squares) agreed well with the calculated values (solid lines) determined using equation (1). The dashed line indicates the minimum *L* obtainable using the 4-mm-long Nd:YAG and RIG film (thickness: 4.75 mm) employed in this study. The pulse shape produced by the cavity with *L* = 10 mm is shown in Fig. 2b. The obtained pulse energy and duration were 27 μJ and 25 ns, respectively, corresponding to a 1.1-kW peak power. The pulse power fluctuated within 7% deviation during more than 10 repeated measurements. To the best of our knowledge, this is the highest peak power value produced to date using MO Q-switches. The output spectrum obtained for *L* = 10 mm was measured using a spectrum analyser (Anritsu, MS9740A) and is shown in the inset of Fig. 2b. There were three peaks in the measured spectrum (black circles). The full widths at half maximum at each centre wavelengths are 47 pm at 1,064.54 nm (red line), 39 pm at 1,064.59 nm (blue line), and 35 pm at 1,064.66 nm (green line). This spectrum split might indicate that there were mainly three modes of propagation beam in the cavity, and the split may be disappear in more shortened cavity. The beam quality *M* ^2^ of the output pulse was also measured using a lens with 25-mm focal length via the knife-edge method, according to ISO Standard 11146^37^. *M* ^2^ was estimated to be 3.7.

### Pumping energy

**Figure 3:**
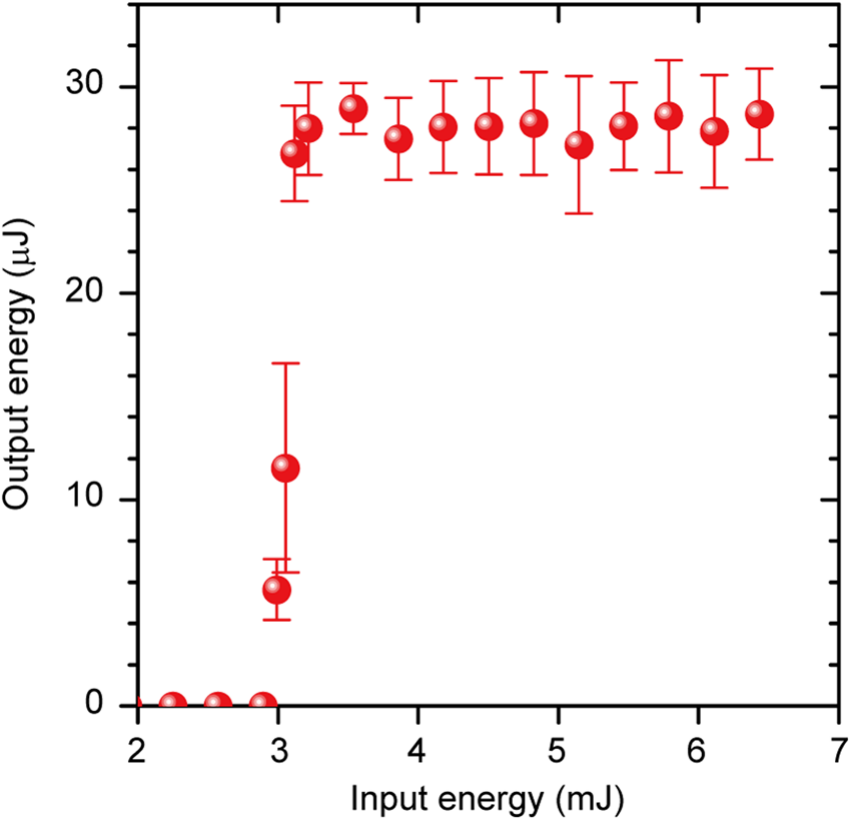
Output energy of magnetooptical (MO) Q-switch laser. Output energy versus pumping energy. The diode peak power was fixed at 32.2 W and the pumping duration was varied from 70 to 200 μs. The pumping-pulse fall time and the magnetic-pulse rise time were set to constants.

To determine the minimum input energy for Q-switching, the pumping energy was changed by modulating the diode-output. The duration between the pumping-pulse fall time and the electric-pulse rise time applied to the coils was fixed to 15 μs. Figure 3 shows the output energy obtained as a function of the input energy for these conditions. The threshold was 2.9 mJ and the output energy became saturated for a pumping energy of more than 3.1 mJ. Such a saturation characteristic shows good agreement with previous experimental reports using passive Q-switch lasers^7^. The output energy is constant until additional pulses are generated. Hence, a single pulse was obtained in this setup.

### Output polarisation

**Figure 4:**
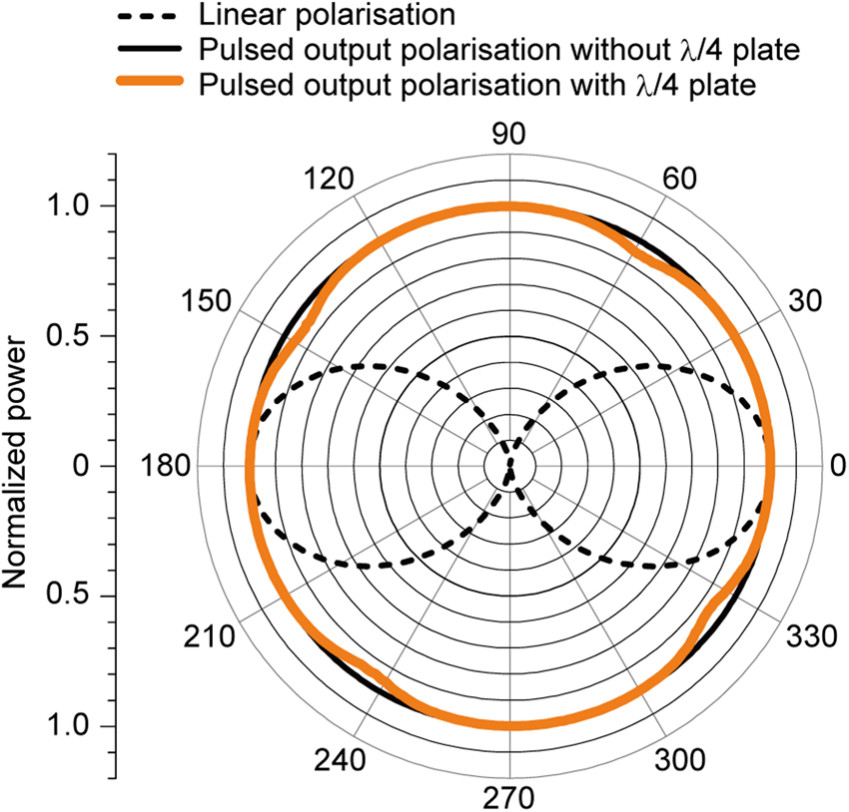
Polarisation state. Output polarisation of Q-switched laser analysed using quarter-wave (*λ*/4) plate and analyser. The ideal linearly polarised state is indicated by the black dashed line. Without the *λ*/4 plate, the transmitted power showed no dependency on the analyser angle (solid black line). The polarisation state using the *λ*/4 plate is plotted by the bold orange line. The polarisation state remained constant with rotation of the *λ*/4 plate, indicating that the output pulse was not circularly but randomly polarised.

The output-pulse polarisation state was analysed using a quarter-wave (*λ*/4) plate and an analyser. Initially, the output power was measured with a rotating analyser, and the results indicated that the state exhibited circular or random polarisation. Then, a *λ*/4 plate was inserted between the output coupler and the analyser. The power change was monitored using an InGaAs-based fast-response optical detector (Thorlabs, DET10C/M). As shown in Fig. 4, the *λ*/4 plate exerted no influence on the polarisation; therefore, random polarisation of the output was confirmed.

## Discussion

In the polarisation state measured using the *λ*/4 plate shown in Fig. 4, small dent-like features are apparent. However, these features were caused by the *λ*/4 plate insertion and are unrelated to the output state produced by the MO Q-switch laser. Note that Nd:YAG has an isotropic crystal structure and emits unpolarised light. Further, although an MO Q-switch using an isotropic lasing material has been reported^38^, this is the first report of a randomly polarised output for an MO Q-switch laser, because the RIG-based MO Q-switch does not contain polarisers. If one needs to control the polarisation state, changing the lasing material from an isotropic crystal to an anisotropic one would be the easiest way. For the laser cavity using Nd:GdVO_4_, which emits linearly polarised light, a circularly polarised output was obtained. Thus, the results obtained in this study show that the MO Q-switch using RIG film can be used with various lasing materials. These results are contrary to the mechanism of an MO Q-switch explained by Faraday rotation. While we do not have a clear model explaining the entire mechanism of the MO Q-switch, these results would help.

Overall, this study demonstrates integrable MO Q-switching using RIG film in an Nd:YAG laser system for the first time. The 10-mm-long cavity produced 1.1-kW peak power and a 27-ns-long output at a centre wavelength of 1064.58 nm via QCW diode pumping, generating randomly polarised pulses. The repetition ratio was 1 kHz. In addition, the output polarisation state was confirmed to be random and an *M* ^2^ value of 3.7 was obtained. In this laser system, the Q-switch and the lasing material have a similar crystal structure; therefore, the MO Q-switch and the Nd:YAG can be combined into actively Q-switched micro lasers, similar to epitaxial growth^31^ or bonding of passive Q-switches on lasing materials (e.g., Cr^4+^:YAG on Nd:YAG)^32,33^. The experimental evidence provided in this study advances this research field toward the realisation of actively controllable integrated micro lasers.
